# Cold-inducible RNA-binding protein (CIRP) potentiates uric acid-induced IL-1β production

**DOI:** 10.1186/s13075-021-02508-9

**Published:** 2021-04-26

**Authors:** Yuya Fujita, Toru Yago, Haruki Matsumoto, Tomoyuki Asano, Naoki Matsuoka, Jumpei Temmoku, Shuzo Sato, Makiko Yashiro-Furuya, Eiji Suzuki, Hiroshi Watanabe, Atsushi Kawakami, Kiyoshi Migita

**Affiliations:** 1 Department of Rheumatology, Fukushima Medical University School of Medicine, 1 Hikarigaoka, Fukushima, Fukushima 960-1295 Japan; 2 Department of Rheumatology, Ohta-Nishinouchi Hospital, 2-5-20 Nishinouchi, Koriyama, Fukushima 963-8558 Japan; 3 Department of Immunology and Rheumatology, Unit of Advanced Preventive Medical Sciences, Nagasaki University Graduate School of Biomedical Sciences, Sakamoto1-7-1, Nagasaki, 852-8501 Japan

## Abstract

**Background:**

Gout is an autoinflammatory disease driven by interleukin-1 (IL-1) induction in response to uric acid crystals. IL-1β production is dependent on inflammasome activation, which requires a priming signal, followed by an activating signal. The cold-inducible RNA-binding protein (CIRP) has been recently identified as a damage-associated molecular pattern (DAMP). In this study, we evaluated the roles of CIRP in monosodium urate (MSU)-mediated IL-1β secretion using human neutrophils.

**Methods:**

Human neutrophils were stimulated by MSU in the presence or absence of CIRP priming to determine NLRP3 inflammasome activation and subsequent caspase-1 activation and IL-1β production. Cellular supernatants were analyzed by enzyme-linked immunosorbent assay (ELISA) to determine the presence of IL-1β or caspase-1 (p20). The cellular supernatants and lysates were also analyzed by immunoblotting using anti-cleaved IL-1β or anti-cleaved caspase-1 antibodies.

**Results:**

Neither CIRP nor MSU stimulation alone induced sufficient IL-1β secretion from neutrophils. However, MSU stimulation induced IL-1β secretion from CIRP-primed neutrophils in a dose-dependent manner. This MSU-induced IL-1β secretion from CIRP-primed neutrophils was accompanied by the induction of cleaved IL-1β (p17), which was inhibited by the pretreatment of MCC950, a specific inhibitor for NLRP3. Furthermore, cleaved caspase-1 was induced in the cellular lysates of CIRP/MSU-treated neutrophils. Additionally, CIRP stimulation induced the protein expression of pro-IL-1β in neutrophils.

**Conclusions:**

Our data indicate that CIRP, an endogenous stress molecule, triggers uric acid-induced mature IL-1β induction as a priming stimulus for NLRP3 inflammasome in human neutrophils. We propose that CIRP acts as an important proinflammatory stimulant that primes and activates inflammasome and pro-IL-1β processing in response to uric acid in innate immune cells.

## Introduction

Gout is the most prevalent acquired autoinflammatory disease characterized by abrupt self-limiting attacks of arthritis caused by the precipitation of uric acid crystals in joints [1]. Recent studies suggest that inflammasome activation and the subsequent IL-1β production play an import role in gouty arthritis [2]. In vitro analysis showed that monosodium urate (MSU) crystal-driven inflammation is dependent on the assembly of the Nod-like receptor pyrin domain containing 3 (NLRP3) inflammasome [3]. Pathogen-associated molecular patterns (PAMPs) or danger-associated molecular patterns (DAMPs), which are thought to serve as ligands for Nod-like receptors, prime the inflammasome and subsequently induce IL-1β production in response to a second activation signal [4]. The production of active IL-1β requires two steps: priming and activation [5]. At the priming step, PAMPs or DAMPs induce the transcription of pro-IL-1β. Subsequently, the primed cells encounter the second activation stimuli, and pro-IL-1β is processed into mature IL-1β by caspase-1 through inflammasome activation [6]. Previous studies demonstrated that no difference is observed in the mRNA level of IL-1β after the MSU crystal-based stimulation of macrophage, suggesting a lack of the priming effect of MSU in inflammasome activation [7]. In line with these findings, MSU itself failed to induce IL-1β secretion [8]. Therefore, it is unclear what priming stimuli trigger gout attacks in patients with hyperuricemia.

The mechanisms that mount innate immune cells on full inflammasome activation may depend on the priming stimuli [9]. DAMPs represent molecules that normally exist within the cells and that are released in the extracellular space upon cellular stress or damage, thus acting as danger signals [10]. In this study, we focused on how uric acid induces pro-IL-1β processing in innate immune cells and thus contributes to the development of gout. Although IL-1β has been identified as a key mediator, the stimulus that primes the inflammasome cascade in a sterile inflammatory arthritis, gout, is unclear [11]. It has been suggested that several DAMPs, which are released by damaged cells, sense the inflammasome as a priming signal [12]. We hypothesized that endogenous molecules released under the conditions of cellular stress [13] sense the uric acid-mediated inflammasome activation. Extracellular CIRP (eCIRP) is a recently discovered DAMP [14]. We report here that CIRP acts as a priming stimulus for inflammasome-dependent caspase-1 activation and IL-1β processing in uric acid-stimulated human neutrophils.

## Materials and methods

### Reagents

Recombinant human CIRP was purchased from Sino Biological (Chesterbrook, PA, USU). The endotoxin level of this recombinant protein is < 1 EU/μg as determined by LAL method. MSU crystals were purchased from Alexis (Lausen, Switzerland). Anti-pro-IL-β Polyclonal antibody (MBS 125139) was purchased from MyBioSource (San Diego, CA USA). Anti-cleaved-IL-β (p17, D3A3Z) antibody was purchased from Cell Signaling Technology (CST, Danvers, USA). Anti-cleaved caspase-1 antibody was purchased from Abcam (Cambridge, UK). Anti-NLRP-3 antibody was purchased from InvivoGen (San Diego, CA USA). NLRP3 Inhibitor, MCC950, was purchased from MERCK MILLIPORE (Billerica, MA USA).

### Neutrophils isolation

Venous peripheral blood were obtained from Japanese healthy subjects (6 males, 1 females, mean age of 34.4 ± 8.7 years). Written informed consent for blood donation was obtained from each individual. The blood was layered on a Polymorphprep TM (Axis-Shield, Oslo, Norway) cushion and neutrophils were purified using density sedimentation according to the manufacturer’s instructions. To determine the effects of CIRP on MSU-induced IL-1β production in neutrophils, freshly isolated neutrophils were pretreated with various concentrations of CIRP for 6 h and then stimulated with MSU.

### ELISA analysis

IL-1β and caspase-1 (p20) amounts in cell-free neutrophil-conditioned media were measured by enzyme-linked immunosorbent assay (ELISA) kits (R&D Systems, Minneapolis) according to the manufacturers’ protocols.

### Cell lysis and immunoblot analysis

Freshly isolated neutrophils were stimulated with CIRP or MSU for indicated periods, and the cells were washed by PBS and added RIPA Lysis Buffer (Sigma-Aldrich) supplemented with proteinases inhibitor cocktail on ice. The cell lysates were centrifuged at 10,000*g* for 10 min at 4 °C and collect the supernatant. An equivalent amount (30 μg) was subjected to 12% SDS-PAGE and electrotransferred onto polyvinylidene fluoride membranes, which were blocked for1 h at room temperature with 5% bovine serum albumin. The membrane was incubated with primary antibodies against human pro-IL-1β, cleaved caspase-1, or β-actin and then incubated with secondary antibodies at room temperature, followed by visualization using ECL reagent (Amersham, Little Chalfont, UK). Immunoblot detection was achieved by LAS-3000 Imaging System (Fuji Film, Tokyo Japan). Neutrophil-conditioned media were also subjected to immunoblot analysis using ant-cleaved-IL-1β antibody.

### Statistical analysis

Differences between groups were examined for statistical significance using Student’s *t* test. *P* values less than 0.05 were considered to be significant.

## Results

### MSU induces IL-1β release from CIRP-primed neutrophils

**Fig. 1:**
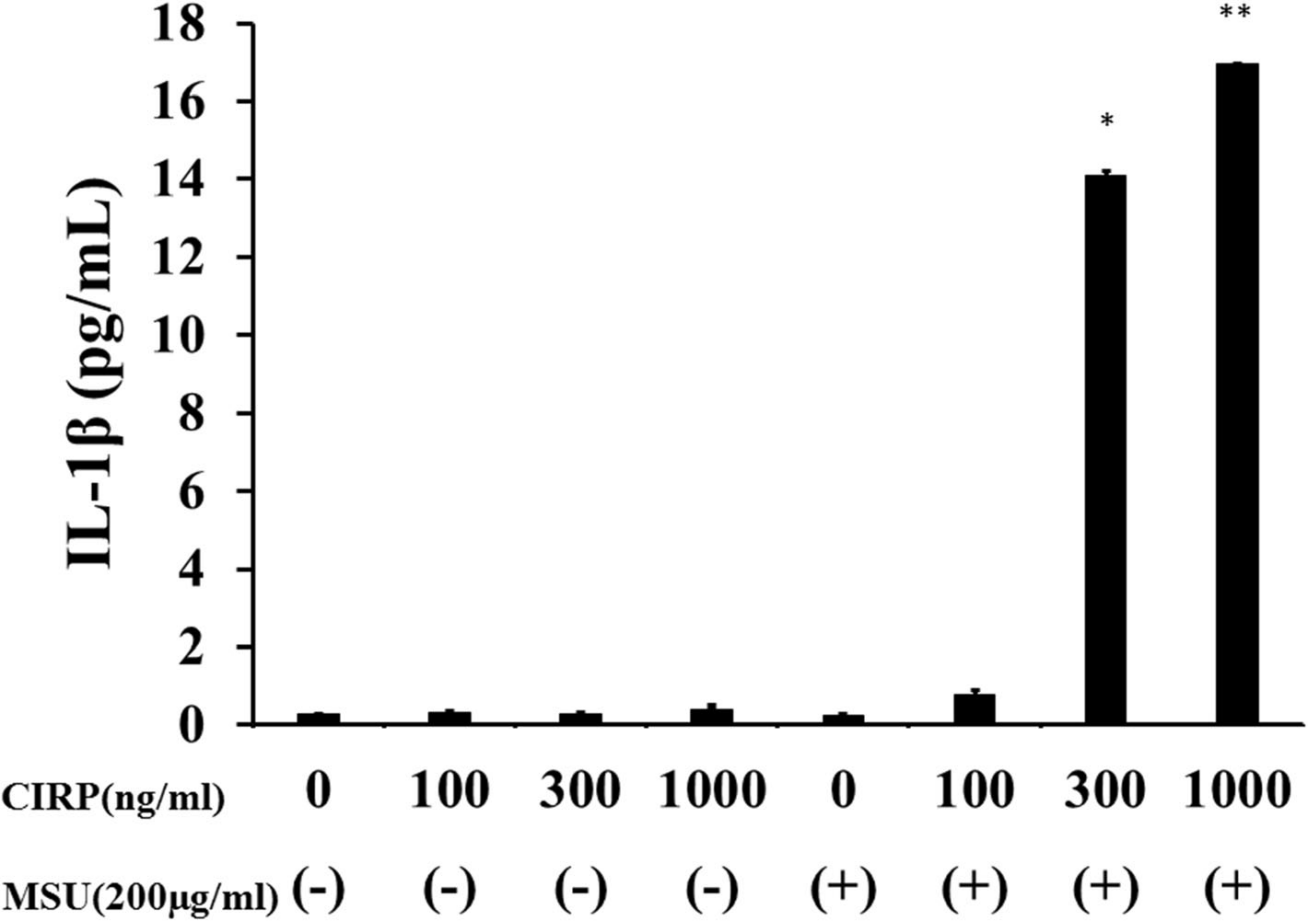
CIRP pretreatment induces MSU-mediated IL-1β synthesis from neutrophils in a dose-dependent manner. Neutrophils (2 × 10^6^/ml) were pretreated with various concentrations of CIRP for 6 h. After pretreatment, the cells were stimulated with or without MSU (200 μg/ml) for 18 h and supernatants were analyzed for IL-1β production by ELISA. Values represent the mean ± SD of two independent experiments. Single asterisk indicates significant difference was observed at a threshold of *p* < 0.01compared to unstimulated control neutrophils. Double asterisk indicates significant difference was observed at a threshold of *p* < 0.001compared to unstimulated control neutrophils

**Fig. 2:**
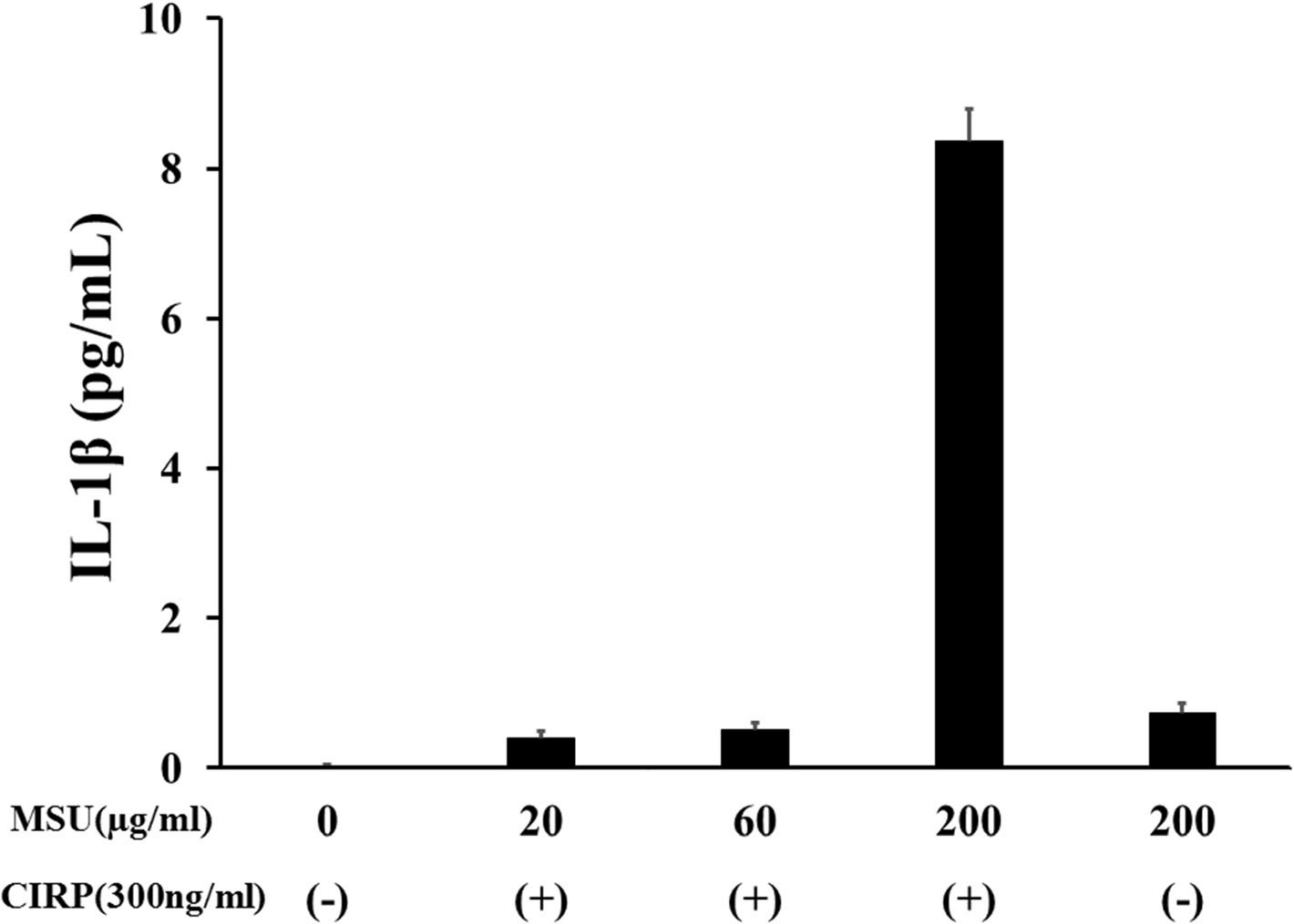
MSU induces IL-1β synthesis from CIRP-primed neutrophils. Neutrophils (2 × 106/ml) were pretreated with various concentrations of CIRP (300 ng/ml) for 6 h. After pretreatment, the cells were stimulated with various concentrations of MSU for 18 h and supernatants were analyzed for IL-1β production by ELISA. Values represent the mean ± SD of two independent experiments

First, we investigated whether CIRP could induce the secretion of IL-1β from neutrophils. Incubation of CIRP did not induce the release of a significant amount of IL-1β from human neutrophils (Fig. 1). Next, we tested the possibility that CIRP acts as a priming stimulus using a model of gouty arthritis, in which uric acid induces IL-1β secretion. Neutrophils were pretreated with CIRP, and at 6 h post-pretreatment, CIRP-primed neutrophils were stimulated with MSU for another 18 h. MSU stimulation alone induced a minimum level of IL-1β release from neutrophils (Fig. 1). However, MSU stimulation induced the release of a substantial amount of IL-1β from CIRP-primed neutrophils in a dose-dependent manner (Figs. 1 and 2).

### CIRP potentiated MSU-induced caspase-1 activation and pro-IL-1β processing

**Fig. 3:**
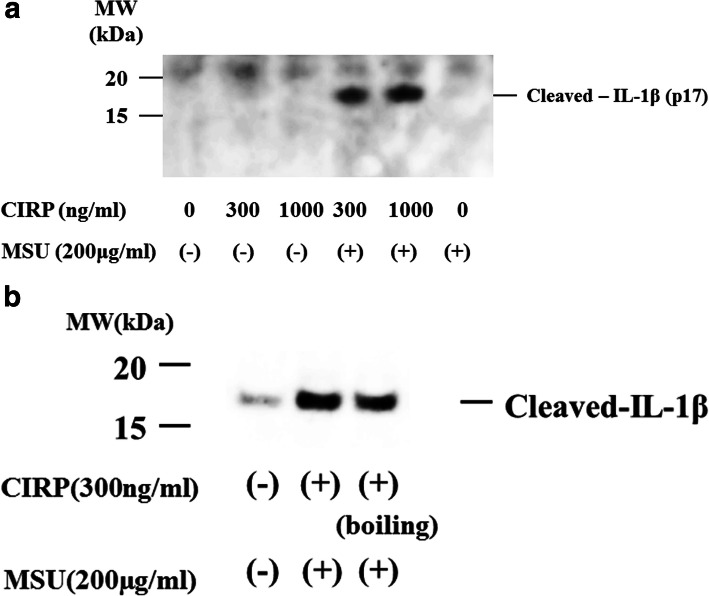
MSU induces cleaved IL-1β secretion from CIRP- primed neutrophils. **a** Neutrophils were pretreated or untreated with the indicated concentrations of CIRP for 6 h. After pretreatment, the cells were stimulated with MSU (200 mg/ml) for 18 h and supernatants were analyzed by immunoblot for the presence of cleaved IL-1β (p17). Three experiments were performed using different neutrophils and a representative result is shown. **b** Neutrophils were pretreated with 300 ng/ml of CIRP or heat denatured CIRP (boiled for 30 min) for 6 h. After pretreatment, the cells were stimulated with MSU (200 mg/ml) for 18 h and supernatants were analyzed by immunoblot for the presence of cleaved IL-1β (p17). Two experiments were performed using different neutrophils, and a representative result is shown

**Fig. 4:**
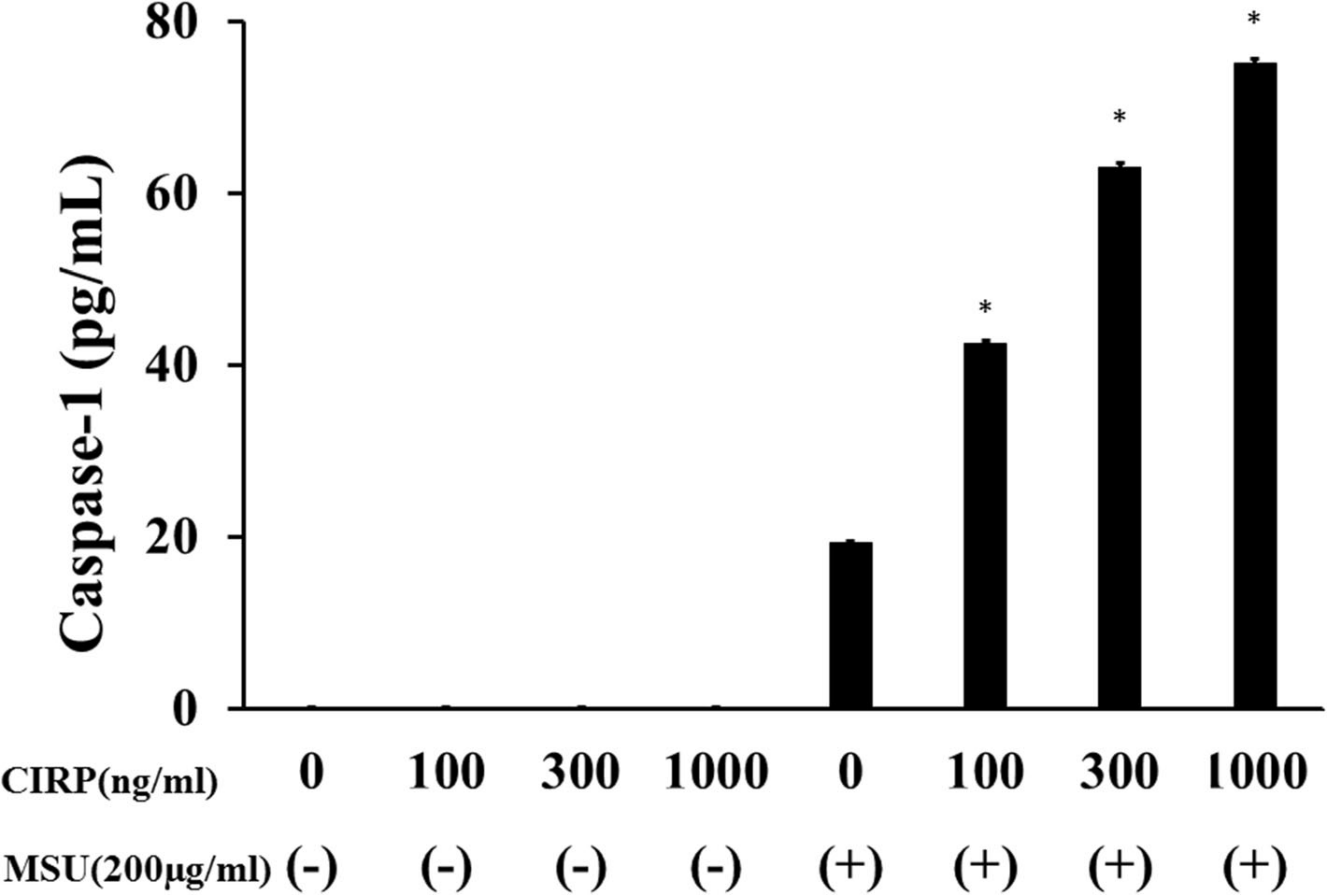
MSU induces caspase-1 (p20) synthesis from CIRP-primed neutrophils. Neutrophils (2 × 10^6^/ml) were pretreated with various concentrations of CIRP for 6 h. After pretreatment, the cells were stimulated MSU (200 μg/ml) for 18 h and supernatants were analyzed for the presence of caspase-1 (p20) by ELISA. Single asterisk indicates significant difference was observed at a threshold of *p* < 0.01compared to unstimulated control neutrophils

**Fig. 5:**
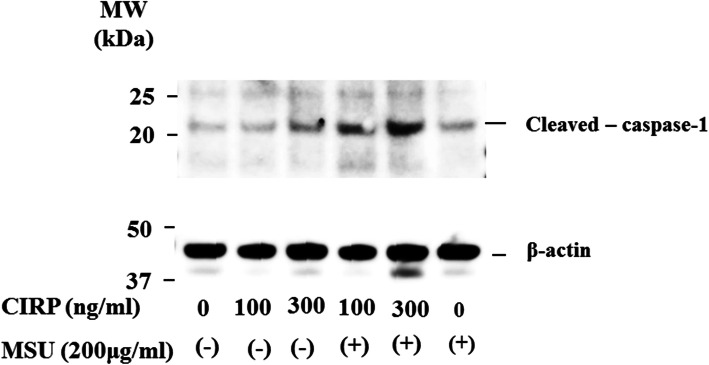
Caspase-1 immunoblot analysis using the cellular lysates of MUS-stimulated neutrophils. Neutrophils were pretreated or untreated with the indicated concentrations of CIRP for 6 h. After pretreatment, the cells were stimulated with of MSU (200 μg/ml) for 18 h. Cellular lysates were analyzed by immunoblotting with antibody that recognize cleaved caspase-1 (p20). β-actin was the loading control. Three experiments were performed using different neutrophils, and a representative result is shown

To examine whether MSU stimulation induces the release of mature IL-1β from CIRP-primed neutrophils, culture supernatants were subjected to immunoblot analysis using anti-cleaved IL-1β (p17) antibody. Stimulation of CIRP-primed neutrophils with MSU induced the production of cleaved IL-1β (p17). However, neither CIRP nor MSU stimulation alone induced cleaved IL-1β production (Fig. 3a). MSU-induced cleaved IL-1β production from CIRP-primed neutrophil was not inhibited by boiling the recombinant CIRP protein for 30 min (Fig. 3b). This result suggested that LPS contamination in CIRP protein preparation was minimal since cytokine-inducing effect is sensitive by boiling for 30 min [15]. In addition to cleaved IL-1β, we examined whether CIRP induced the cleaved caspase-1 (p20), which was processed from pro-caspase-1 via inflammasome activation [16]. To investigate whether CIRP priming leads to the activation of pro-caspase-1, cleaved caspase-1 (p20) was measured by caspase-1 (p20)-specific ELISA. MSU stimulation alone did not induce the release of cleaved caspase-1 from neutrophils. By contrast, MSU stimulation induced the release of cleaved caspase-1 from CIRP-primed neutrophils in a dose-dependent manner (Fig. 4). We also determined pro-caspase-1 activation by immunoblot analysis using cellular lysates from CIRP/MSU-treated neutrophils. Figure 5 shows the increase in the protein levels of the cleaved caspase-1 p20 subunit in CIRP-primed neutrophil cell lysates after stimulation with MSU. However, neither CIRP nor MSU stimulation alone induced cleaved caspase-1 release in the neutrophil cell lysates.

### Effects of MCC950 on MSU induces IL-1β release from CIRP-primed neutrophils

**Fig. 6:**
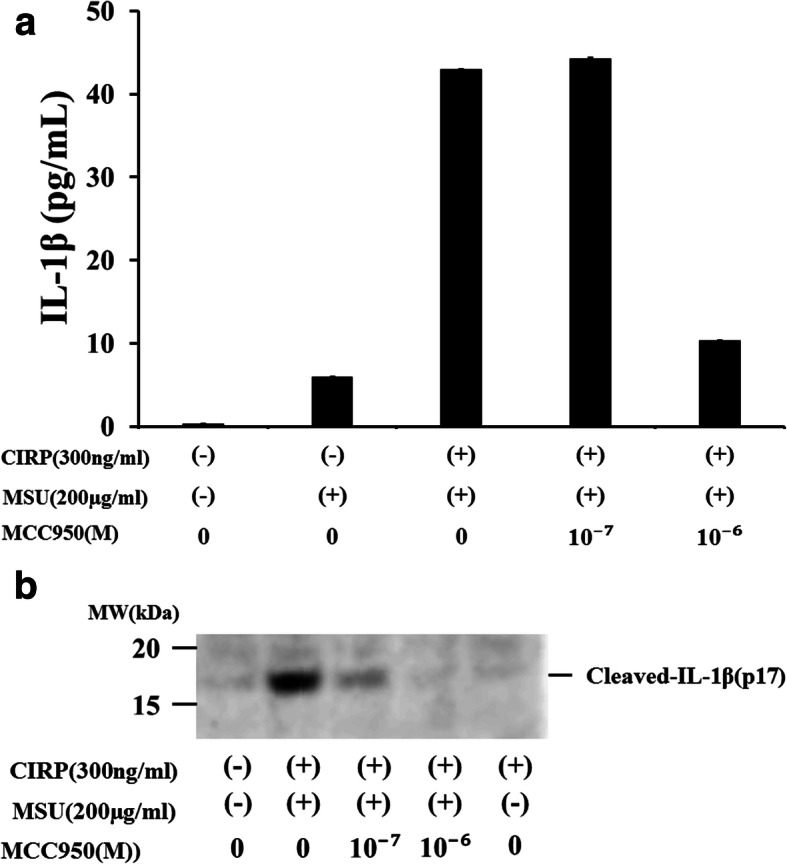
**a** Effects of MCC950 on MSU-induced IL-1β synthesis from CIRP-primed neutrophils. CIRP (300 ng/ml, for 6 h)-primed neutrophils were pretreated with or without MCC950 for 30 min, and then stimulated with MSU (200 μg/mL) for 18 h. Supernatants were analyzed for IL-1β production by ELISA. Values represent the mean ± SD of two independent experiments. Significant difference was observed at a threshold of *p* < 0.01 between CIRP/MSU-stimulated neutrophils with and without pretreatment of MCC950 (10^− 6^ M). **b** Effects of MCC950 on MSU-induced cleaved-IL-1β (p17) expression of CIRP-primed neutrophils. CIRP (300 ng/ml, for 6 h)-primed neutrophils were pretreated with or without MCC950 for 30 min, and then stimulated with MSU (200 μg/mL) for 18 h. Cellular lysates were analyzed by western blotting using anti-cleaved-IL-1β antibodies. Two experiments were performed using different neutrophils, and a representative result is shown

**Fig. 7:**
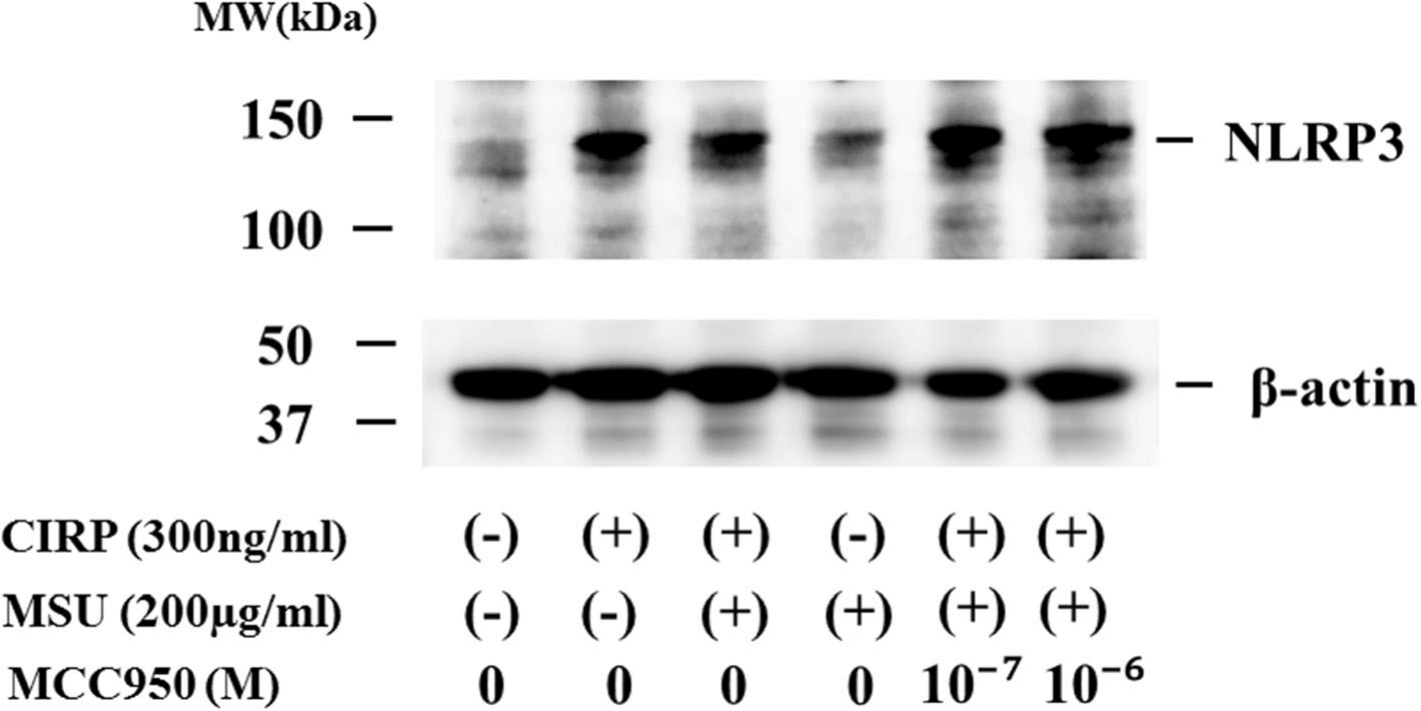
CIRP-induced NLRP3 expression in neutrophils. CIRP (300 ng/ml, for 30 min)-primed neutrophils were pretreated with or without MCC950) for 30 min and stimulated with MSU (200 μg/mL) for 18 h. Cellular lysates were analyzed by western blotting using anti-NLRP3 antibodies. β-actin was the loading control. Two experiments were performed using different neutrophils, and a representative result is shown

MCC950 is a highly specific small molecule inhibitor of NLRP3 inflammasome [17]. We examined whether MCC950 inhibit the activation of pro-IL-1β by inhibiting the activation of NLRP3 inflammasome in CIRP/MSU-stimulated neutrophils. As shown in Fig. 6, inhibition of IL-1β release was observed in neutrophils treated with MCC950 at 1 μM and stimulated with MSU (Fig. 6a), whereas the inhibition of the release of cleaved IL-1β (p17) was partially observed in neutrophils treated with MCC950 at 0.1 μM and completely observed in those with MCC950 at 1 μM (Fig. 6b). We also checked the protein expression of NLRP3, a major component of the inflammasome complex, in neutrophils. In unstimulated neutrophils, NLRP3 protein expression was barely detected and marginal expression of NLRP3 was induced by MSU stimulation, whereas its expression was increased in response to CIRP pretreatment or CIRP plus MSU stimulation. MCC950 did not affect this enhanced NLRP3 protein expression of CIRP/MSU-stimulated neutrophils (Fig. 7).

### CIRP induces pro-IL-1β expression in neutrophils

**Fig. 8:**
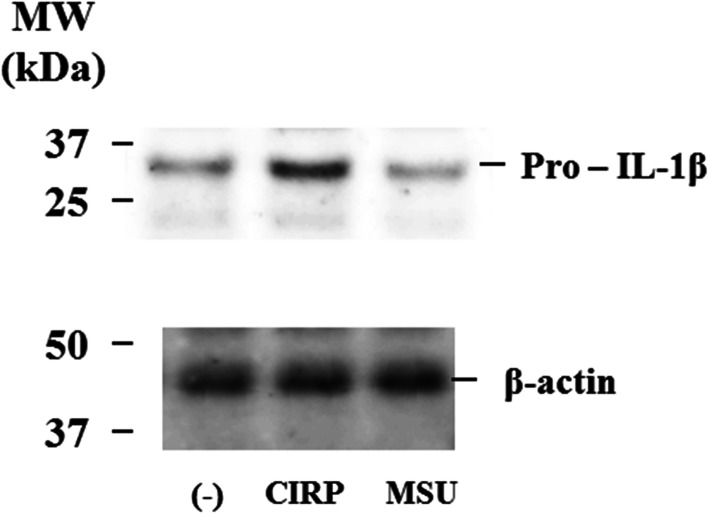
Pro-IL-1β immunoblot analysis using the cellular lysates of CIRP or MSU-stimulated neutrophils. Neutrophils were pretreated or untreated with CIRP (300 ng/ml) or MSU (200 μg/ml) for 18 h. Cellular lysates were analyzed by immunoblotting using anti-pro-IL-1β antibody. β-actin was the loading control. Three experiments were performed using different neutrophils, and a representative result is shown

To determine whether CIRP can have a direct effect on neutrophils, we stimulated neutrophils with CIRP and investigated whether CIRP treatment induced the expression of pro-IL-1β protein by immunoblot analysis. Figure 8 shows a significant increase in the protein levels of pro-IL-1β in the cell lysates of CIRP-treated neutrophils.

## Discussion

It has been postulated that the release of active IL-1β needs a first signal that induces the pro-IL-1β gene transcription, a process described as “priming,” followed by a second signal that activates the inflammasome, resulting in caspase-1 activation and subsequent IL-1β processing [18]. This information, together with the observation of neutrophil invasions around the uric acid crystals in gouty arthritis [19], suggests that neutrophils function as danger sensing cells that contribute to the production of biologically active IL-1β. Neutrophils do not always require lipopolysaccharide (LPS) priming under sterile conditions in contrast to the well-documented “two-hit-model,” in which LPS may act as a constant phase of alarm during cellular stress [20]. The mechanism underlying inflammasome activation by uric acid in the absence of PAMPs, such as LPS, has not been elucidated [6]. Increased serum and synovial fluid levels of CIRP, one of the endogenous DAMPs, were reported in patients with inflammatory arthritis [21, 22]. We speculate that CIRP can sense MSU-mediated inflammasome activation in innate immune cells. In this study, we assessed the role of CIRP as a priming stimulus for uric acid-induced inflammasome activation and pro-IL-1β processing using human neutrophils.

To study the effects of CIRP on neutrophils, we isolated human peripheral blood neutrophils and primed these cells with recombinant human CIRP, followed by MSU stimulation. Consistent with previous reports [23], MSU stimulation alone did not result in sufficient IL-1β secretion from human neutrophils. However, CIRP-pretreated neutrophils produced mature IL-1β in response to the subsequent MSU stimulation. These data indicate that CIRP possesses proinflammatory properties, which enable it to prime the neutrophils for full inflammasome activation in response to MSU stimulation. Our data clearly indicated that the stimulation of neutrophils with MSU alone did not activate caspase-1 and mature IL-1β production. To investigate the ability of CIRP to sense neutrophils, we investigated pro-IL-1β expression in neutrophils. Our results indicated that CIRP stimulation alone induced an increase in the protein level of pro-IL-1β in neutrophils. By contrast, MSU stimulation alone did not alter the pro-IL-1β protein levels in neutrophils. This suggests that, mechanistically, CIRP induces pro-IL-1β expression, which could be needed for full inflammasome activation of and IL-1β secretion following MSU stimulation. CIRP employs numerous receptors to stimulate immune responses [24]. Although we did not elucidate the putative receptor, CIRP elicits the proinflammatory cascades by upregulating the pro-IL-1β mRNA or protein level, which appear to be involved in mature IL-1β secretion in response to uric acid in human neutrophils. Thus, CIRP seems to be one of the DAMPs that promote full inflammasome activation and processing of IL-1β in uric acid-mediated autoinflammatory cascades.

Overall, this study elucidates a novel mechanism that explains how CIRP primes the inflammasome and pro-IL-1β processing following MSU stimulation. eCIRP exposure can be an important priming signal for MSU-mediated autoinflammation via the upregulation of the pro-IL-1β protein level. DAMPs are endogenous self-molecules released by the cell under stress or upon damage [25]. Priming the inflammasome through interaction with DAMPs has been demonstrated in the gouty arthritis model [26]. We hypothesize that an endogenous stress molecule, CIRP, possesses inflammasome priming effect and triggers pro-IL-1β processing in response to inflammasome-activating stimuli.

## Conclusion

We showed that an endogenous stress molecule, CIRP, primes MSU-mediated caspase-1 activation and the subsequent pro-IL-1β processing as a priming signal. These data suggest that stress signals, including CIRP, could trigger uric acid-mediated inflammasome activation and pro-IL-1β processing. Characterizing the involvement of CIRP in various inflammatory disorders is critical for the identification of the pathophysiological processes of autoinflammation.

## Data Availability

Not applicable

## Abbreviations

CIRP: Old inducible RNA-binding protein
DAMPs: Danger-associated molecular patterns
IL-1: Interleukin-1
LPS: Lipopolysaccharide
MSU: Monosodium urate
NLRP3: NLR family pyrin domain containing 3
PAMPs: Pathogen-associated molecular patterns
